# Doctors in Fiction

**Published:** 1887-07-30

**Authors:** 


### Doctors in Fiction.

(*Concluded from p. 279, vol. ii.*)

Something of what Lydgate might have been, as well as something which his too severely intellectual nature could not be, is shown in "Dr. Levier," the New Orleans doctor, whom the American novelist Cable has lately depicted. Dr. Levier loves his work much; but he loves his patients more. Physiology and psychology go hand in hand in his studies of human nature, and philanthropy unites and dominates the two. The story of his life and work is like a comment on Longfellow's words—

"Talk not of wasted affection; affection is never wasted."

The love which might, in apparently happier circumstances, have been concentrated on wife and children, flows out to all who come near him, and strews his path with endless blessings.

Another American writer, Howells, gives us in "Dr. Breen's Practice" one of the new complications of medical life. Dr. Breen is a woman, a young and pretty one; and though she has duly "walked the hospitals," and taken a degree at Philadelphia University, the only patient she ever had is her mother, who falls ill of some trifling, in great part imaginary, complaint, when the two are staying at a seaside hotel. At first Mrs. Breen is satisfied with her daughter's attendance, but when she has invented a more serious symptom she insists on having a male medical attendant. Grace, her daughter, accordingly requests the doctor of the neighbouring village to meet her in consultation; and here the difficulty occurs. For the doctor is old-fashioned in his ideas. He admires Miss Breen greatly; he even falls in love with her, and subsequently asks her to be his wife; but he will not recognise Dr. Breen's medical pretensions, and positively refuses to consult with her. The end of it is that Grace is compelled, in order to satisfy her mother, to give up the charge of the case to the male doctor; and in despair she throws up medicine altogether and marries a rejected suitor of her pre-medical days, who opportunely turns up at the time.

The situation is decidedly comical, but an undercurrent of sadness somewhat belies the laughter: the disappointment of any unselfish and worthy ambition is always sad. The same characteristic of tears behind smiles marks a little known story of Jules Sandeau, called "Le Docteur Herbeau." The hero of this is an elderly and somewhat grotesque physician, who cherishes a sentimental but very reverent passion for a young lady who lives near him. Her home is uncongenial, and she suffers from a languor and melancholy which the doctor in vain essays to cure. Finally, there comes upon the scene a rival practitioner, young and handsome, who restores the damsel to health by simply falling in love with her. The last scene in the story depicts the old doctor dying—of nothing in particular, doubtless: for of course old folks never suffer from heart-break—and seeing the lady of his worship ride past his open window with her lover, while her gay laugh—the laugh of youth, and health, and happiness—is borne to his ears on the summer air.

A very uncanny personage is the doctor in Lytton's "Strange Story," whose experiments are only less terrible in their results than the miraculous transformation in Stevenson's "Dr. Jekyll and Mr. Hyde." Mr. Hyde, as most people know, is Dr. Jekyll, or rather all the evil in Dr. Jekyll's nature concentrated by the power of a miraculous drug he has invented in another personality. Dr. Jekyll is to those who know him in the world a bland, courteous, well-behaved gentleman; he takes a glassful of his medicine and becomes Edward Hyde, a dwarfed, misshapen creature, delighting in all the evil a conscienceless will can invent; another glassful, and he is the doctor once more. A pleasant, if dangerous power this, if it could be controlled; but it cannot. One morning the doctor finds that he has changed into Mr. Hyde in his sleep, and after that time the transformations became frequent and erratic. The power of evil is gaining strength; no mysterious medicine is required to bring Mr. Hyde into existence now; the slightest cessation of the action of conscience, the faintest shadow of a sinful thought, and he springs into being, while continually increasing doses are required to make the doctor himself again. At last the magic drug is exhausted, and he cannot obtain the means of making more. Changed without hope of reform into Edward Hyde, the doctor kills himself to escape the sinful and degraded existence which lies before him if he remains in that personality.

It is a weird, wonderful, ghastly story; and though the physical transformation depicted in it is, as my friend said, "quite impossible," none of us can look within and fail to perceive that the soul does indeed contain a dual personality, a nature that struggles towards good and a nature that tends to evil, and can be kept subdued only by continual watchfulness.

This is the latest notable portrait added to the gallery of doctors in fiction; but now that some of the best known members of the profession are finding time to write novels, we may hope that some very worthy and complete medical pictures will be added to the collection.

